# Modelling of Human Low Frequency Sound Localization Acuity Demonstrates Dominance of Spatial Variation of Interaural Time Difference and Suggests Uniform Just-Noticeable Differences in Interaural Time Difference

**DOI:** 10.1371/journal.pone.0089033

**Published:** 2014-02-18

**Authors:** Rosanna C. G. Smith, Stephen R. Price

**Affiliations:** 1 Department of Cell and Developmental Biology, University College London, London, United Kingdom; 2 Centre for Mathematics, Physics and Engineering in the Life Sciences and Experimental Biology (CoMPLEX), University College London, London, United Kingdom; University of Salamanca- Institute for Neuroscience of Castille and Leon and Medical School, Spain

## Abstract

Sound source localization is critical to animal survival and for identification of auditory objects. We investigated the acuity with which humans localize low frequency, pure tone sounds using timing differences between the ears. These small differences in time, known as interaural time differences or ITDs, are identified in a manner that allows localization acuity of around 1° at the midline. Acuity, a relative measure of localization ability, displays a non-linear variation as sound sources are positioned more laterally. All species studied localize sounds best at the midline and progressively worse as the sound is located out towards the side. To understand why sound localization displays this variation with azimuthal angle, we took a first-principles, systemic, analytical approach to model localization acuity. We calculated how ITDs vary with sound frequency, head size and sound source location for humans. This allowed us to model ITD variation for previously published experimental acuity data and determine the distribution of just-noticeable differences in ITD. Our results suggest that the best-fit model is one whereby just-noticeable differences in ITDs are identified with uniform or close to uniform sensitivity across the physiological range. We discuss how our results have several implications for neural ITD processing in different species as well as development of the auditory system.

## Introduction

The ability to localize sound sources accurately is critical to the survival of many species and also contributes to the human ability to follow conversations in noisy environments, the so-called “cocktail party effect” [1]. In order to achieve this, binaural comparisons of several different features of the sound are made, as first observed by Lord Rayleigh [2]. In the azimuthal plane intensity differences caused by a head shadowing effect are the major cue used by humans for source localization for high sound frequencies (over 2 kHz). Below 1 kHz, these intensity differences are much lower and so sound localization is dominated by comparison of timing differences at each ear, so called interaural time differences (ITDs) which are based on the detection of interaural phase differences (IPDs). Brughera et al. [3] have demonstrated that humans are sensitive to ITD fine structure in sound up to a limit of 1.4 kHz. The split of localization cue usage into two broad frequency ranges is known as the duplex theory [2], [4] and is most closely adhered to for pure tones [4]–[6]. For broadband sounds the situation is more complex, with ITDs contributing as a localization cue at higher frequencies [7], [8]. However, the contribution from ITDs to sound localization is very small for high frequencies [9] and our analysis of ITD sensitivity concerns low frequencies (below 1.4 kHz). Additionally, there is also some variation between individuals in their cue-usage [10]. Human speech uses frequencies in the low frequency range, with fundamental frequencies of approximately 130 Hz for men and 220 Hz for women and first formants for vowel discrimination below 1000 Hz [11]–[13]. Use of this low frequency range can also be observed in sung vocalization, where the high note of a soprano is roughly 1000 Hz and a low note by a bass singer is approximately 100 Hz. Hence, use of the low frequency region means that ITDs are the main cue for sound localization in human vocal communication. Other animals use these interaural comparisons in different frequency regions, depending on head size and cue sensitivity.

Several factors affect the magnitude of ITDs including the distance separating the two ears (related to head size in most animals), sound frequency and azimuthal position of the sound source. For all head sizes and frequencies, a sound produced at the midline reaches each ear at the same time, assuming symmetrically placed ears across the midline. ITD increases as the sound source is positioned at greater azimuth angles. For humans, a sound located at one side of the head (90° azimuth) generates a maximum ITD of around 750 µs for low frequency sounds [14]. Although midline ITD is minimal, the rate of change of ITD signal with angle is greatest at the midline and humans and other animals are known to be best at localizing sounds from this location. The sensitivity with which humans localize a sound is known as auditory acuity or minimal audible angle (MAA). MAA is a relative measure of localization ability and is the just-noticeable difference (jnd) in sound angle. MAA displays a non-linear variation with azimuthal angle for humans [15], [16] and experiments in barn owls have also uncovered a similar variation in their localization ability [17]. In both cases, acuity was around 1° at the midline, increasing to around 10° laterally. Angular variation of acuity depends upon two factors when sound is localized using an interaural comparison cue [15]. These factors are the variation of localization cue with azimuth angle and the sensitivity of cue identification. Angular variation of ITD has been established using both experimental measurements and modelling studies [14], [15], [18], [19]. However, sensitivity of ITD identification depends on the auditory processing system and is less well characterized. ITD sensitivity is considered to be the just-noticeable difference in ITD (jnd ITD) and can encompass both precision and accuracy errors. As discussed later, when accuracy errors are low, ITD jnds are a measure of precision of ITD identification [20].

There is some inconsistency in the literature concerning the variation of jnd ITD with angle. It is sometimes attested that poor acuity out at the sides (90°) is due to poor lateral ITD sensitivity arising from fewer neurons detecting long ITDs [21]–[23]. However, it has been observed in birds that detection of ITDs by brainstem neurons is equally spread across the ITD range. There, the neural circuit for detection of the ITD cue uses coincidence detection of bilateral synaptic input to the nucleus Laminaris to produce a map of ITDs and hence sound location [24]–[27]. In that circuit, longer ITDs are represented equally to shorter ITDs [28], [29]. There may be species differences in the sensitivity with which ITDs are detected over the physiological range, just as there are clear differences in how ITDs are coded within the auditory brainstem [1], [30]. Some studies have looked directly at the sensitivity of ITD identification, observing just-noticeable differences in perception of the ITD cue for pure tones [31] and for broadband sounds [32]. For the human pure tone data of Domnitz and Colburn [31], there are only a few data points across the physiological ITD range. However, these data points have been interpreted either as evidence for near constant sensitivity across the ITD range [33] or as evidence of significantly poorer sensitivity at longer ITDs compared to short ITDs [31]. Hafter and de Maio [34] also conducted measurements of jnds ITD using a broadband, click stimulus. Their results demonstrated a slight gradual increase in ITD jnds when ITD ranged from 0 µs to 500 µs. Although this range is a significant proportion of the maximum physiological human ITD, it only accounts for approximately half the azimuth range, as 500 µs ITD corresponds to approximately 45° azimuth for frequencies below 1 kHz. The aim of this study is to gain further insight into the issue of how jnds ITD vary with ITD magnitude and the overall contribution they make to sound localization acuity.

Acuity is dependent on both ITD sensitivity and the rate of change of angle with ITD. The rate of change of angle with ITD increases as azimuth angle increases, meaning that the same change in ITD corresponds to a larger change in angle at more lateral azimuths. Acuity displays a similar overall variation, with larger MAA values at greater azimuths. We explore the extent to which acuity variation is influenced by the dual factors of ITD sensitivity and the angular variation of ITD. Our analysis makes no assumptions about the neural procedure for identifying ITD and hence the results concerning sensitivity of ITD identification are characteristic of the entire ITD processing system rather than a single part of the auditory pathway.

This study uses previously published data on azimuthal variation of auditory acuity between 0° and 90° by Mills [15] and Schmidt et al. [16]. In those studies a psychometric function was determined using a forced-choice method for constant stimuli in which tones were presented in pairs and subjects were asked whether the test sound was located to the left or right of a reference tone. Acuity values (MAAs) were evaluated as half the difference between 25% and 75% points on the psychometric function. In order to utilize the available acuity data we firstly investigated the most appropriate acoustic model for calculating interaural comparisons (ITDs and ILDs) for the experimental set up in those studies. Secondly, we compared ITD predictions from the acoustic model to empirical measurements and determined which acuity data sets could be analysed using the acoustic model. We then calculated ITD jnds for individual acuity data points and identified possible distributions for ITD jnds across the ITD range. Finally, the candidate distributions were used to find best-fit acuity curves for the original acuity data sets. Our results are most compatible with uniform, or near uniform just-noticeable differences in ITD identification, increasing the evidence in this direction from other studies. A similar, but approximate, approach was used by Kuhn [35] to indicate that Mills' acuity values could be compatible with predicted acuity if ITD sensitivity is assumed to be constant. However, the acoustic model used by Kuhn was simplified and did not allow for frequency dependence or non-negligible sound source distance, unlike the acoustic model used in this study. Additionally, we perform a rigorous analysis of the acuity data to both suggest and test potential distributions of jnds in ITD. This leads us to an explanation of how auditory objects at central angles are localized with greater sensitivity as a direct consequence of non-linear ITD variation with sound source location, which dominates over the angular variation of just-noticeable differences in ITD. This further piece of evidence for uniform sensitivity of ITD identification has implications for sound localization acuity in other species and development of the ITD processing system.

## Methods

**Figure 1:**
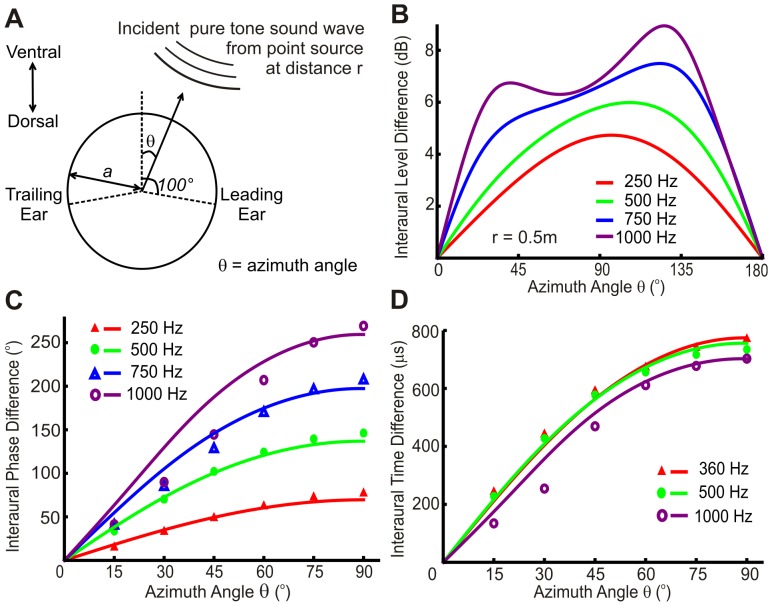
Model predictions for human ILDs and ITDs. **A**, Model to determine ITD or ILD variation with azimuth angle θ for the experimental set up in Mills [15], Schmidt et al. [16] and Kuhn [14]. The human head is modelled as a solid sphere, radius a (8.75 cm) and the sound source is modelled as a point source, frequency f, and distance r from the centre of the head. Ears are positioned 100° away from the midline. **B**, Azimuthal variation of interaural level difference (ILD) for sound source at 0.5 m, 250 Hz, 500 Hz, 750 Hz or 1000 Hz, as predicted by our acoustic model for the experimental set up by Mills. **C**, Comparison of predicted curves for interaural phase differences (IPDs) and empirical data points from Mills [15], r = 0.5 m. **D**, Comparison of model interaural time differences (ITDs) with empirical data from Kuhn [14], r = 3.0 m.

The acuity data from Mills [15] and Schmidt et al. [16] are for pure tone sound sources at distances of 0.5 m and 1.35 m respectively. These distances are not quite far enough to consider the incident sound as a plane wave. Therefore, to calculate ITD variation with azimuth angle, we used the Rabinowitz [18] model based on the Rschevkin solution [36] to determine the pressure on the surface of a sphere from a point sound source (form S_f_e^−2πift^) at distance r. We modelled the head as a sphere of radius a, with ears set back at 100° from the midline [37] as shown in Figure 1A. The radius of an adult human head is taken as 8.75 cm in all analyses [38]. Angular frequency is ω = 2πf and wave number is k = (2πf)/ν where f is frequency and ν is speed of sound in air (340 ms^−1^ based on an ideal gas at 15°C). The head-related transfer function, H, relates the pressure that would be present in the free field, P_freefield_, to the pressure developed at the surface of the sphere, P_surface_.
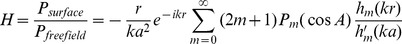
(1)


P_m_cos(A) are Legendre polynomials of order m and argument cos(A). Angle A is the angle in radians between a ray from the centre of the sphere to the sound source and a ray from the centre of the sphere to the measurement point on the surface. When considering the pressure at the two ears for a sound source at azimuth angle θ relative to the midline, A = 100°−θ for the leading ear and A = 100°+θ for the lagging ear. h_m_(kr) are spherical Hankel functions (first kind), order m and argument kr. h'_m_(ka) are the derivatives of the spherical Hankel functions, argument ka. The head-related transfer function, H, is a function of k, a, r and θ and is a complex wave with phase Φ.
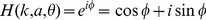
(2)


The phase for a particular k, a, r and θ can be found using the argument of H:
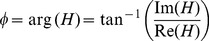
(3)


By evaluating H at the leading ear (H_+_) and at the lagging ear (H_−_) we were able to determine the IPD for a given incident sound angle:
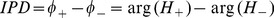
(4)


This allowed us to calculate the interaural time difference (ITD) for a given frequency of sound from the relation:
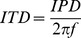
(5)


Our evaluation of the head-related transfer function used a maximum m value of 6. The difference in predicted ITDs using an m_max_ value of 5 and m_max_ of 6 is less than 1 µs over the whole 0°<θ<180° range for a sound of 1000 Hz at a distance of 0.5 m and is less than this for lower frequencies or greater sound source distances. The interaural level difference (ILD) in decibels was calculated from the difference in magnitude of the head related transfer function at each ear:
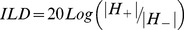
(6)


For calculations of acuity, the rate of change of azimuth angle was determined using the following identity for total derivatives, which is valid when derivatives are continuous and non-zero and therefore can not be used at exactly 90° or 270°.
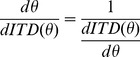
(7)


All calculations for model predictions and best-fit comparisons to human data were performed using Wolfram Mathematica 7.0.0, Wolfram Research Inc, Champaign, IL, USA. Best-fit curves were determined using the NonLinearModelFit function for a least-squares fit of data points to constant, linear or non-linear functions. Best-fit curves were calculated using regression analysis, determining best-fit parameters to a confidence level of 95%. Variation of angle θ with ITD in the time domain was determined using numerical interpolation of data points of the angle at which a given ITD occurs. Adjusted R^2^ and Corrected Akaike Information Criterion (AICc) values were calculated for best-fit solutions. AICc values are a measure of the relative quality of a statistical model for a given set of data and are founded on estimates of the information lost when a given model is used to represent the process that generates the data [39]. AICc values are used for acuity model comparisons as these are non-linear functions for which adjusted R^2^ values can not be used as a goodness of fit measure.

Comparative predictions of acuity for human newborns and adults were calculated for a distant 500 Hz sound source, ΔITD = 15 µs and head radius for newborns calculated from World Health Organization child growth standards [40], taking the average of girl and boy 50^th^ percentile values for head circumference.

## Results

Auditory acuity depends on both angular ITD variation and ITD sensitivity (jnd ITDs). We used the data on the variation of human acuity with azimuth angle from two studies, Mills [15] and Schmidt et al. [16], for pure tone sounds at 250 Hz, 500 Hz, 750 Hz and 1000 Hz (Mills) or 500 Hz and 1000 Hz (Schmidt). The Mills study displays acuity data in two figures (5 & 6). We consider the data obtained from the figures both separately and combined in this study. However, the acuity data from the Mills study and the Schmidt study are not combined due to their different experimental set ups. Firstly, we found the most appropriate acoustic model for ITD variation in the Mills and Schmidt experiments. We then determined the acuity data for which ITD is used as the localization cue. Subsequently, we used the acoustic model to calculate ITD jnds (ΔITDs) for each of the individual acuity data points and investigated their distribution.

We used a standard and well-regarded model of the head as a solid sphere, radius 8.75 cm, with ears at 100° away from the midline. Although this is not an exact physical model for a head it is a good analytical approximation and one that has been shown to be a close approximation for ITD variation at low frequencies around a morphologically human mannequin head [41]. In both the Mills and Schmidt data, the incident sound is a pure tone from a point source at distance r from the centre of the head. These distances were 0.5 m for the data from Mills and 1.35 m for the data from Schmidt et al. Interaural time differences (ITDs) or interaural level differences (ILDs) were calculated by evaluating the argument or magnitude respectively of the head-related transfer function (H) at each ear (eqns 4,5 & 6). We calculated the head-related transfer function (H) using the solution by Rabinowitz et al. [18] (reproduced by Duda and Martens [19]), for a point sound source of theform S_f_e^−2πift^ where S_f_ is a frequency-dependent amplitude (**Figure 1A**). The acoustic model we employed is appropriate for the experimental data and we retain the frequency dependence of ITD by calculating the head-related transfer function to a high degree of accuracy, in comparison to the frequency-independent Kuhn approximation [42].

We aimed to investigate the sensitivity of ITD identification and therefore only used the acuity data for which ITD could reasonably be asserted to be the dominant sound localization cue. To determine which acuity data sets were suitable, we investigated whether ILDs were significant in the acuity studies, or whether ITDs dominated in sound source localization. It has been demonstrated that ILDs as well as ITDs are above threshold detection in the low frequency region when the sound source is close to the head [19], [41]. To investigate whether this is the case for the experiments in Mills and Schmidt et al., angular variation of ILD and ITD were calculated for a sound source 0.5 m away, at 250 Hz, 500 Hz, 750 Hz and 1000 Hz (**Figure 1B**), as for the Mills data. ILD is a monotonic function of azimuth at 250 Hz and 500 Hz, similar in form to IPD and ITD azimuthal variations (**Figure 1C,D**). At 750 Hz and 1000 Hz the ILD variation displays significant changes in gradient around 45° azimuth, markedly different to ITD variation which does not display this significant change in gradient. The sensitivity threshold to ILD detection in humans is of the order of 0.5–1.5 dB [43]. We found that ILD magnitude is greater than this sensitivity threshold for the majority of azimuth positions and could therefore act as a sound localization cue in the Mills experiment. ILD variation across the azimuth range (approximately 6 dB at 500 Hz) is of the same order of magnitude as the sensitivity of ILD detection (approximately 0.5–2 dB [31], [43]). However, the ratio of ITD range to sensitivity is approximately 75∶1 (755 µs range at 500 Hz and 10–20 µs jnd ITD [31]). Therefore, it can be seen that localization is dominated by the ITD based component. In addition, Wightman and Kistler [44] have demonstrated that location judgements are perceptually dominated by ITDs at low frequency when both ITD and ILD cues are present. For the Schmidt data, ITD domination of sound source localization is an even better approximation as the extra distance between the head and sound source (1.35 m) reduces ILDs at all angles and frequencies. However, owing to the non-monotonic variation of ILD above 750 Hz as well as other factors described below, ITD sensitivity was investigated using the available acuity data at 250 Hz and 500 Hz only.

**Figure 2:**
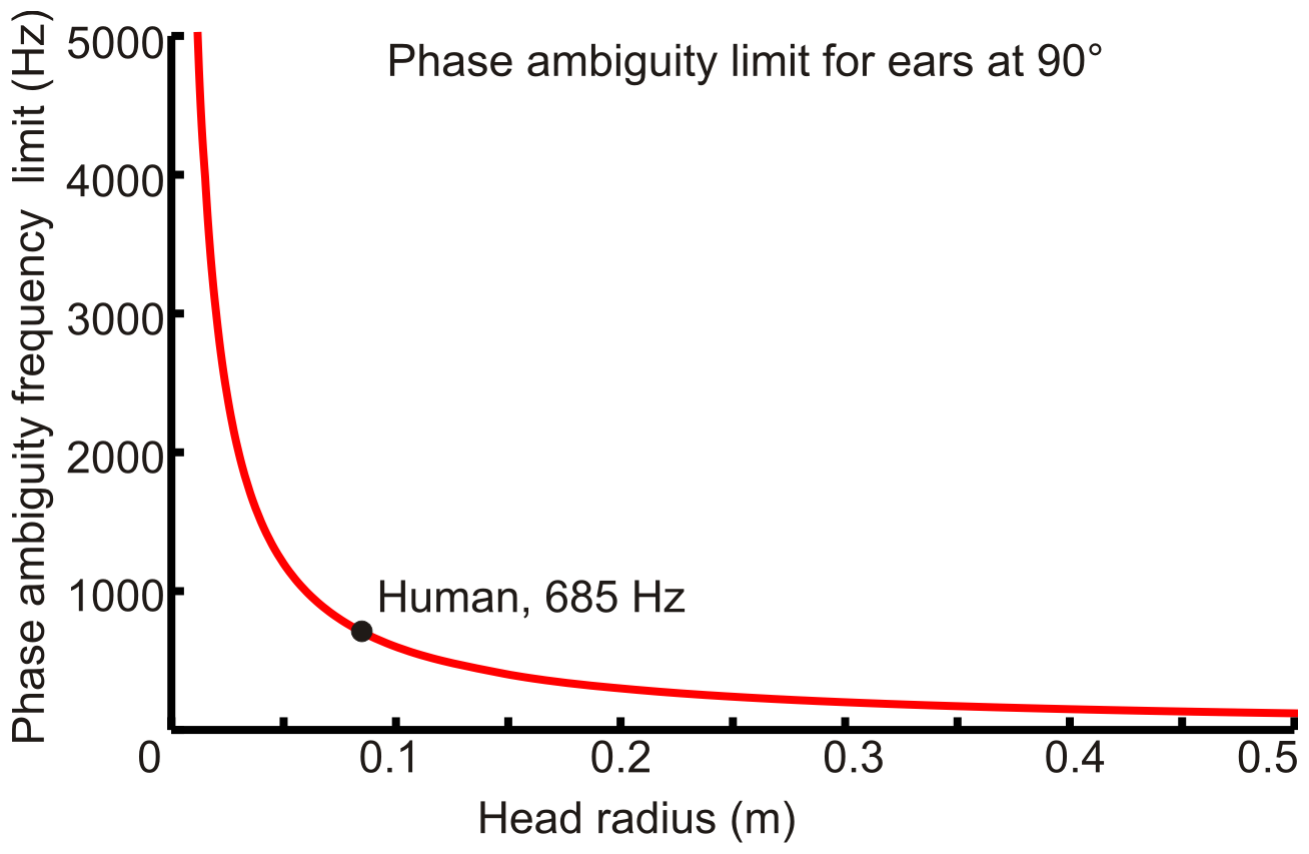
Phase ambiguity frequency limit for varying head size. This is the frequency at which interaural phase difference first reaches 180°. This model is for ears at 90° away from the midline. In this case the human phase ambiguity limit is 685 Hz (695 Hz for ears at 100°) for a head radius of 8.75 cm. As head size increases animals are restricted to using IPD as a non-ambiguous sound localization cue at lower frequencies. Animals with smaller heads have a greater range of frequencies in which IPD is a non-ambiguous cue.

To test our model of angular ITD variation we compared IPDs and ITDs measured by Kuhn [14] and Mills [15] respectively to IPD and ITDs predicted by our model. We found a good fit between empirical and modelled values at and below 500 Hz, but above 500 Hz they diverge from each other in the 30°–60° azimuth region (**Figures 1C,D**). This divergence could be due to variables not included in our model such as non-spherical head shape, which has been shown to influence ITDs in a frequency-dependent manner for mid-range azimuth angles [41]. Additionally, we excluded the use of acuity data at 750 Hz and 1000 Hz as these frequencies lie above the phase ambiguity limit. The phase ambiguity limit is the frequency at which the IPD first reaches 180° at any azimuth angle [45]. Above this frequency there is ambiguity as a given IPD could correspond to a sound source from more than one angle. Although the methodology used in Mills and Schmidt studies are such that confusion between the hemispheres is less likely as relative position (left/right) rather than absolute position is ascertained [45], it is possible that phase ambiguity could have affected the resultant acuity data. Using our ITD model, the phase ambiguity limit is predicted to be at 695 Hz. Further predictions of the phase ambiguity limit for different head sizes are shown in **Figure 2**. As head size decreases, the phase ambiguity limit increases so animals are able to use IPD and hence ITD as a localization cue up to higher frequencies. We concluded that ITDs can be considered as the dominant sound localization cue for the Mills and Schmidt acuity data at or below 500 Hz and we restricted further analysis to this frequency range. We also concluded that there is a good fit between ITD data at these frequencies and predictions of ITD using our acoustic model. These conclusions allowed us to model ITD variation with sound source angle and hence how the decrease in the rate of change of ITD with azimuth angle contributes to the increase in MAA values with angle.

**Figure 3:**
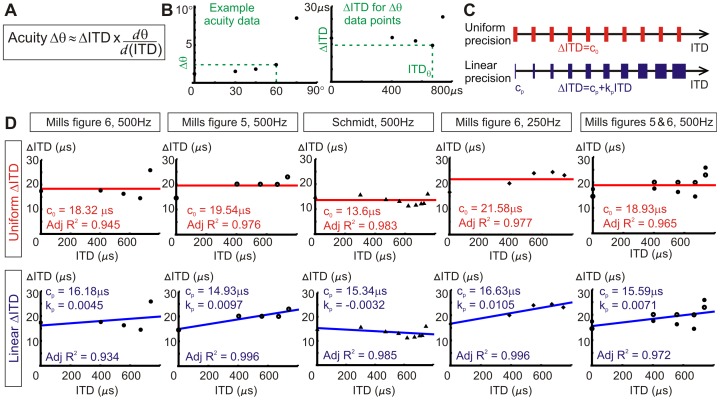
Predicted values of ΔITD determined from acuity data. **A**, Relationship between localization acuity (Δθ), just-noticeable difference in ITD identification (ΔITD) and the angular variation of ITD. **B**, Example of how ΔITDs are determined for each individual data point in an acuity data set. **C**, Candidate models for ΔITD distributions across ITD. Uniform distributions are described by parameter c_0_ and linear distributions are described by parameters c_p_ and k_p_. **D**, Best-fit uniform or linear distributions for each acuity data set under consideration. Overall goodness of fit is similar for both distributions. Midline predictions of ΔITD are better for the linear distribution, but the proportionality constant is low in all cases and negative in one case (Schmidt data), making them close to the uniform case.

Acuity, the sensitivity of relative sound localization (Δθ), is dependent on two factors; jnds in ITD (ΔITD) and the rate of change of angle with ITD. The latter can be calculated using our acoustic model. Our first approach used acuity data and the rate of change of angle with ITD to consider the potential variation of ΔITDs. Using the relationship shown in **Figure 3A**, each acuity data point was considered separately and a value for ΔITD determined. As shown for sample data in **Figure 3B**, these ΔITDs were plotted against the predicted ITD from our model for the corresponding angle. We observed that predicted ΔITDs for individual acuity data points could potentially be described as either a uniform distribution of ΔITD across available ITDs or as one where ΔITD varied linearly across the range. These two candidate distributions are shown schematically in **Figure 3C** and it is assumed that only one distribution is implemented across the physiological ITD range. We considered what the effect on acuity would be if ΔITD was inversely proportional to ITD (smaller ΔITDs for larger ITD magnitudes). In that case, localization acuity would be poor at the midline, best between 30° and 60° and increasingly poor again towards 90° (data not shown). As this is contrary to observed human behaviour it was not considered further.

The best-fits for uniform or linear model distributions are shown in **Figure 3D** for the 5 different data sets. The uniform distributions all slightly overestimate ΔITD at the midline, except for the Schmidt data set. The best-fit linear distributions are better at accounting for midline ITDs. However, the overall goodness of fit values are very similar for the two candidate distributions, which is indicated by similar adjusted R^2^ values. The proportionality constants (k_p_) for best-fit linear distributions are low, ranging between 0.0045 and 0.011 for the Mills data. These k_p_ values relate to differences in ΔITD of only 3.4 µs and 8.3 µs respectively, for sounds at 0° and 90° azimuth. We also observed that the magnitude of our predicted ΔITDs are of a similar magnitude to the just-noticeable differences in ITD found by Dominitz and Colburn [31], demonstrating broad agreement between an experimental approach to determine ΔITDs and our modelling approach.

**Figure 4:**
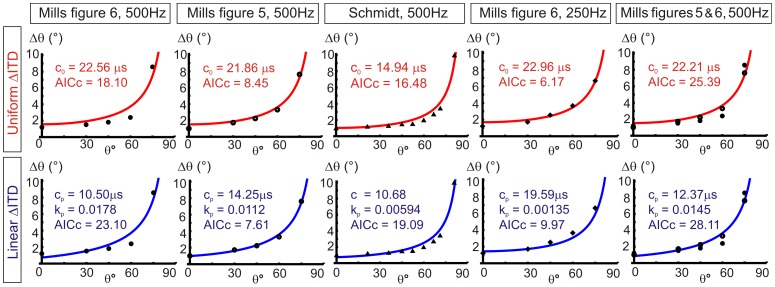
Best-fit acuity curves for uniform or linear ΔITD distributions. Candidate uniform and linear ΔITD models used to find best-fit acuity (Δθ) distributions for five acuity data sets. Uniform distributions are described by parameter c_0_ and linear distributions are described by parameters c_p_ and k_p_. As with the best-fit descriptions of ΔITD distributions, best-fit acuity distributions have low k_p_ values for the linear ΔITD case, close to the uniform case. AICc values indicate that the majority of acuity data sets are more appropriately described by uniform ΔITD distribution as these have lower AICc values than for a linear ΔITD distribution (comparing the same data sets).

**Figure 5:**
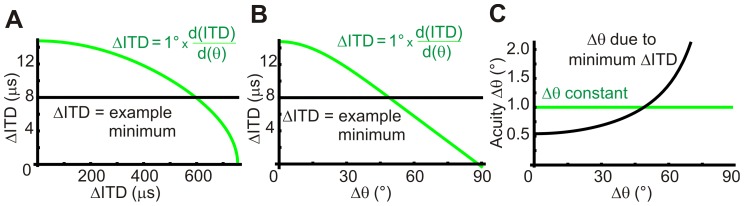
The effect of minimal available ΔITD on acuity. Just-noticeable differences in ITD identification (ΔITDs) required to produce the same acuity (1°) at all angles, shown for the time domain (**A**) and angular domain (**B**). This is compared to an example of the minimum possible ΔITD available in the ITD identification system (across all ITDs). At some point across the range, the minimum ΔITD is greater than the ΔITD required for constant acuity, leading to poorer acuity laterally than at the midline (**C**). The crossover point depends both on the maximum sensitivity of the system (minimum ΔITD) and the value of constant acuity.

Both uniform and linear ΔITD candidate distributions were tested against the original acuity data. Best-fit acuity curves for uniform or linear distributions are shown in **Figure 4** along with their parameters and corrected Akaike information criterion values (AICc). Our predicted acuity values rise steeply towards infinity around 90°, where ITD is maximum (our idealised acuity is discontinuous at ITD_max_). Again, we found that the best-fit linear jnd ITD distributions have low proportionality constants and are essentially close to the uniform case. AICc values were used to evaluate how well the non-linear acuity models account for the data. The value of AICc is used to compare different models for the same data set, a lower AICc value indicates a better explanation of the data by the model. The AICc calculations take into account the the number of parameters in a model, thus including a measure of model complexity in order to prevent overfitting of data. We used the corrected version of AIC owing to the low number of data points per data set. We found that all of the data sets except one (Mills figure 5) have lower AICc values for the uniform ΔITD best-fit curves than for the linear ΔITD best-fit curves. This indicates that uniform ΔITD is a slightly more appropriate model for ΔITD variation than linear ΔITD as it accounts for the data without introducing unnecessary complexity. Linear ΔITD best-fit curves also have very low proportionality constants, essentially making them close to the uniform model.

Our results demonstrate that uniform or near uniform ITD jnds lead to poor lateral acuity compared to the midline. We next asked whether constant acuity across all angles was hypothetically possible for any binaural listener. We reasoned that any physiological system is subject to a limit in ΔITD that relates to the maximum available sensitivity of the ITD processing system across all ITDs. In essence, it is not possible to have zero ΔITD, ie. infinitely good sensitivity. We calculated the variation in ΔITD that would be required to produce a constant acuity at all angles (**Figure 5**). We found that in both the time domain and angular domain the required ITD jnds decrease to zero for sounds located at 90°. Thus, the minimum available ΔITD will become greater than the ΔITD distribution producing constant acuity at some point in the 0° to 90° range. In terms of acuity, this suggests that acuity due to a limit in ITD processing will dominate at lateral angles even if a processing system attempts to produce constant acuity across all angles. This limitation of poor lateral acuity for binaural pure tone detection is presumably overcome by a listener's ability to move their head and place the sound object closer to the midline and hence towards best acuity. The form of the lower bound on acuity would be different if the angular variation of ITD changed. For example, the presence of large pinnae results in deviation of ITDs from those predicted by a spherical head model [46]–[49].

Overall, the non-linear angular variation of ITD creates a lower bound on acuity for a given head size and minimum ΔITD (sensitivity limit of ITD identification). These analyses also demonstrate that the dominant component in human pure tone localization acuity is due to the variation of ITD with angle and that the sensitivity of ITD identification by the processing system is uniform or close to uniform across the ITD range for low frequency sounds.

## Discussion

We have taken an analytical approach to determine the most appropriate description of just-noticeable differences in ITD identification for pure tone sounds. Our analyses are based on using angular acuity data where ITD can be considered as the dominant interaural cue for the sound localization task. Our results are most consistent with uniform or near uniform ITD jnds to describe previously observed psychophysics of human pure tone source localization. Additionally, we determined that there is a limiting form of acuity variation due to the maximum sensitivity available to any binary ITD processing system. This limiting, lower bound on acuity has the same form as for uniform jnd in ITD, with good acuity at the midline which is fairly constant out to 60° but then becomes increasingly poor for more lateral pure tone sound sources.

Our prediction of uniform or near uniform ITD jnds is a property of the whole ITD processing system and is consistent with the ITD jnds found by Dominitz and Colburn [31]. Although our approach is not a direct measurement of ITD just-noticeable differences it adds insight into the role they play in sound localization on two accounts. Firstly, any human neural processing model of auditory information for sound localization does not require the system to be less sensitive at processing long ITDs in comparison to shorter ones. The variation of ITD as a localization cue results in poor lateral acuity compared to the midline when all ITDs are identified with the same level of sensitivity. Secondly, no matter what neural processing strategy is adopted, all binaural listeners are subject to a limit in their ability to identify an ITD. For listeners where the head can be modelled as a sphere, without large pinnae, the limit of acuity takes a similar form to that shown for humans. This minimum available ΔITD may be lower than the actual ΔITD for any given interaural time difference, but it results in a lower bound on acuity which dominates for lateral angles.

Our analysis required us to ascertain which acuity data sets were suitable for determining ITD jnds and led to us rejecting frequencies higher than 500 Hz owing to the likelihood of ILD as a significant localization cue and the additional potential complications of the phase ambiguity limit in source localization. An additional consideration we made in relation to the acuity data was whether the data could be used as a measure of sensitivity of angle identification. This requirement highlights the differing effects of accuracy and precision errors. Accuracy errors affect the ability of a listener to determine the absolute location of a sound source and can be manifested as bias in the identified angle of a sound source. In humans [50] and owls [51], there is evidence of a bias towards the midline for sound source angles around 90°. This behaviour may be influenced not only by ITD identification but also by cues such as spectral shifting by the pinnae, which allows discrimination between front and back [52]. In contrast, precision errors are an indication of the spread around the perceived sound source location for repeated localization attempts. The acuity data used in this study are minimum audible angles, which are measures of the ability to discriminate the relative location of two sound sources and hence are just-noticeable differences in sound location. Previous studies have reasoned that minimal audible angles are a measure of localization precision [53]–[55]. However, it has been shown by Moore et al. [20] that this relationship is only valid when accuracy errors are small. As we do not have information regarding the accuracy of location judgements for the data sets from Mills or Schmidt et al., we regard the MAAs in this study as just-noticeable diffferences in angle and hence our conclusions concern just-noticable differences in ITD and could be a combination of precision and accuracy errors in ITD identification.

How do constant just-noticeable differences in ITD relate to processing of the ITD signal? Our conclusion that ITDs are processed with uniform jnds results from an analytical model that encompasses the entire auditory system, irrespective of how ITDs are actually coded within the nervous system. Just-noticeable differences in ITD identification are often used as a behavioural outcome to test models of binaural neural processing, which is one of the reasons for undertaking this study. Early models for binaural neural comparisons required the processing system to have fewer nerve fibres encoding for long ITDs [21], [56]. This distribution of binaural-comparison detectors was needed in order for model outcomes to agree with tone-in-noise experiments and has also been used as weighting variable in straightness and centrality models [22], [23]. However, experimental studies have demonstrated a different distribution of ITD-sensitive neurons in the inferior colliculus of the cat [33], [57] and guinea pig [58], with a greater number of neurons out towards long ITDs, including some outside of the physiological range. A binaural comparison model incorporating this mammalian distribution [33] predicts pure tone just-noticeable ITD differences to be almost constant across ITD when neural processing depends on phase differences as an independent variable rather than time differences. Phase difference dependence is also demonstrated in the study by McAlpine et al. [58]. When IPDs instead of ITDs are considered as the binaural comparison cue, our results are qualitatively the same, with the most appropriate model of ΔIPD as uniform across IPD, but varying in magnitude between 250 Hz and 500 Hz.

**Figure 6:**
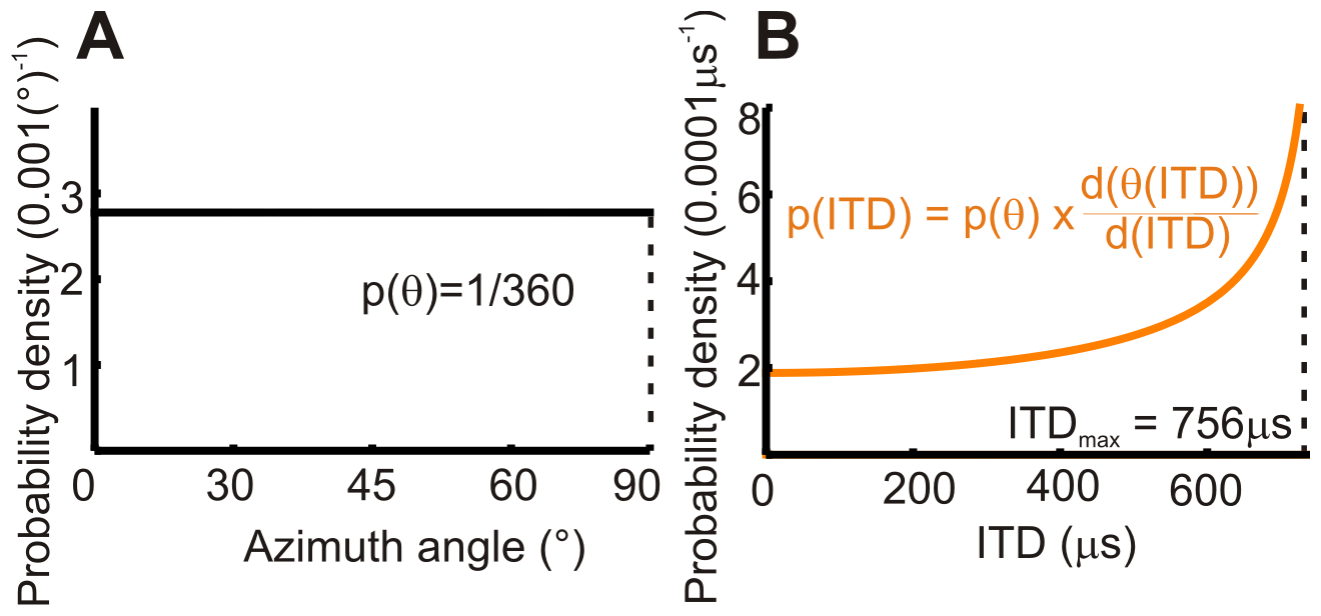
Probability distributions for a sound source in the angular and time domains. Probability distributions in the angular (**A**) and time (**B**) domains for a distant pure tone 500 Hz sound with equal probability of originating at any azimuth angle. This results in a non-constant probability distribution across ITDs, with a greater probability of a sound having a long ITD.

Investigations into the most appropriate model for binaural neural processing have also demonstrated a unifying principle across species, that “the ITDs an animal encounters should be coded with maximal accuracy” [59]. This principle results in processing strategies that vary across species and across frequencies for a single species in order to maximize the information available. Across-species comparisons have also utilized over-arching principles such as the “lower envelope principle” [60], [61] to explain variations and similarities of ITD and ILD cue-usage and localization ability. It should be noted that the analytical model we used to determine ITD variation with azimuth angle can be used to find the probability distribution of encountering any given ITD. The probability distribution for ITD is often required in neural processing models and **Figure 6** demonstrates that if there is an equal probability of a sound coming from any angle, the probability of any given ITD increases as its magnitude increases. Overall, our results suggest that predictions from human neural processing models should result in uniform or near uniform ITD sensitivity for low frequency, pure tone stimuli.

Within the brainstem of birds, constant ITD jnds are suggested by physiological recordings of uniform, equally spaced ITD tuning curves in the nucleus Laminaris [28], [29]. Our results therefore also predict that if avian ITD jnds are constant across ITDs then acuity variaton will take the same form across ITDs as shown for humans. However, it is possible that higher-order processing in the auditory pathway may result in deviation from constant ITD jnds. For owls, which rely heavily on sound localization for nocturnal hunting, studies have been carried out on both localization behaviour as well as the discriminatory properties of the midbrain auditory space map. Sound localization ability is known to be similar to humans, with best acuity at the midline, becoming increasingly poor out towards 90° [17], [51]. Further acuity observations with pure tone stimuli and models of owl ITD variation with angle, would be required to use the methodology presented here to determine whether owls are similar to humans and operate with constant sensitivity of ITD identification. Bala et al. have conducted experiments that assess the discriminatory performance of the auditory space map at the midbrain level of auditory processing [62], [63]. In order to determine whether jnds ITD are uniform at this level of processing would require extension of that assessment across the whole ITD range.

Sound localization acuity would be expected to show variability with head size through head size dependence of ITD. Consideration of head size raises some interesting questions concerning the plasticity of auditory acuity during development. As an individual matures and its head size increases, the maximum ITD increases. The angular variation of ITD has been experimentally determined for several different animals through their development, including cat [48], chinchilla [64] and feret [46]. Those studies demonstrate that the rate of change of ITD with angle (ITD slope) around the midline increases during maturity. If just-noticeable differences in ITD are assumed to be constant across the ITD range then measurement of the ITD slope can be used as an indicator of spatial acuity. The experimental measurements of ITD during development agree with predictions from our model that even if the sensitivity of ITD identification does not improve during neural processing development, increased head size results in better midline acuity as an animal matures.

The effect of head size on sound localization during human development can be seen by comparing maximum ITD and acuity between newborns and adults. Maximum ITD is 483 µs for a newborn (5.44 cm head radius) compared to 755 µs for an adult (see **Methods**) and the plasticity needed to accommodate this change in ITD representation of azimuth can be seen from our prediction that newborn maximum ITD corresponds to 38° azimuth for an adult. Studies in owl [65] have demonstrated plasticity in the auditory processing system during head development and we postulate that plasticity would also be required for humans in approximately the first 30 months of life, during which the head reaches 95% of adult size [64], [66]. Plasticity of the neural system would be expected to affect jnds ITD, which would in turn affect localization acuity. However, the change in head size also affects angular ITD variation, resulting in a decrease in midline MAA from 1.7° at birth to 1.07° as an adult (constant jnds ITD of 15 µs for both newborn and adult). These predictions demonstrate how our method of acuity analysis could be used for further investigation of jnds ITD and plasticity during human and animal development.

We have demonstrated that the most appropriate model for human sensitivity of ITD identification (just-noticeable differences in ITD) is one that is uniform or near-uniform for all physiological ITDs at a single low frequency. The non-linear angular variation of ITD creates a lower bound on acuity for a given head size and greatest available ITD sensitivity. Our results show that acuity towards 90° is always predicted to be worse than at the midline, whatever the neural basis for ITD processing.
